# Computer-tailored smoking cessation advice matched to reading ability: Perceptions of participants from the ESCAPE trial

**DOI:** 10.1016/j.pec.2015.06.013

**Published:** 2015-12

**Authors:** Kirsty Bennett, Hazel Gilbert, Stephen Sutton

**Affiliations:** a Department of Primary Care and Population Health, University College London, London, UK; b Behavioural Science Group, Institute of Public Health, University of Cambridge, Cambridge, UK

**Keywords:** Smoking cessation, Computer tailoring, Literacy, Perceptions, Self-help

## Abstract

**Objective:**

To explore perceptions of computer-tailored advice reports for smoking cessation matched to the recipient's reading level.

**Methods:**

Current cigarette smokers in the UK aged 18–65 who completed a Smoking Behavior Questionnaire (*n* = 6911) were randomized to receive standard generic materials or standard materials plus computer-tailored reports adapted to the recipient's reading level. Smoking status and perception of the reports was assessed at a 6-month follow-up. 4677 participants were included in the analysis.

**Results:**

53.3% were categorized into the easy reading group (ERG). The relative benefit of the intervention for prolonged 3-month abstinence was more marked in the ERG (2.6%/1.9%, OR = 1.50) than in the standard reading group (SRG) (4.0%/3.8%, OR = 1.05), although the interaction was not statistically significant. Participants in the Intervention group perceived the standard materials more positively than did those in the Control group, and participants in the ERG perceived both the generic material and the tailored report more positively.

**Conclusions:**

The easy reading version of this brief self-help intervention was better perceived than the standard version, and appeared to have a small, but promising effect in smokers with a lower literacy level.

**Practice implications:**

An association between reading level and deprivation emphasizes the need to adapt smoking cessation materials to address the needs of smokers with lower literacy.

## Highlights

• Many smokers do not have high literacy skills.
• Self-help materials tailored to reading level showed some benefit for lower literacy smokers.
• The easy reading version of tailored self-help was better perceived than the standard version.
• Smoking cessation materials should address the needs of smokers with lower literacy.

## 1 Introduction

Smoking is a major public health issue and although the number of smokers is in decline, 20% of adults aged 16 and over in England continue to smoke, and 79,000 deaths in 2011 could be attributed to smoking [1]. There is a social gradient in smoking, 26% of adults in manual occupations smoke, in comparison to 15% of adults in non-manual groups [2].

The Skills for Life Survey [3] found that 15% of UK adults aged between 16 and 64 performed at Entry Level 3 or below in literacy which is equivalent to National School Curriculum attainment at age 9–11. This equates to around 5.2 million adults in the UK with a reading age of 11 or lower. As these lower level literacy skills are associated with socioeconomic deprivation [4], it is likely that there is some overlap with the gradient in smoking prevalence with social class.

Self-help materials are available as an aid to quitting. Although there are a number of advantages of generic written self-help materials [5], the effect of written self-help materials on smoking cessation is small [6]. However, Kreuter, Strecher & Glassman [7] suggested that the ‘one size fits all’ approach used in printed health education cannot address the diverse needs of different individuals and proposed the case for computer-tailoring in health education materials. Computer based systems can be used to generate advice reports individually tailored to a smoker's characteristics, for example motivation to quit, dependence and previous quit attempts, and can be produced at a low cost and delivered on a large scale [8]. Research has found that computer-tailoring for individual smokers has a small but useful impact on smoking cessation [6], [9], [10].

Additionally, research has found that self-help materials for smoking cessation are often written at a level beyond the skills of many readers. Meade & Byrd [11] found that the mean reading level of self-help booklets for smoking cessation was grade 10.5 (age 15/16) but 54% of participants had reading levels less than grade 6 (age 12). Smokers from lower social grades make fewer quit attempts [12], and if self-help materials are beyond the skills of low literacy individuals, the gradient in smoking with social class may widen. Thus an important feature of health promotion literature is that it is written at an appropriate reading level. This can be achieved with individual computer-tailoring, where materials can also be tailored to the education and reading level of the individual [13]. Qualitative work exploring smokers’ perceptions of computer-tailored feedback reports adapted to different levels of readability suggested that materials written at the level of the participant would be acceptable, but also that the level must take account of more educated recipients, who would feel patronized if sent materials designed for a lower reading level [14].

The ESCAPE study was a randomized controlled trial that compared the effectiveness of personal computer-tailored advice reports with standard self-help materials [13]. The study aimed to recruit a representative sample consisting of smokers with varying levels of motivation, readiness to quit and dependency, and of all levels of reading ability. The unique feature of the ESCAPE trial in comparison to other studies investigating computer-tailoring in health information, was that the smoking cessation advice reports were tailored to an individual's reading ability. In this paper we:

1. Describe differences in participant characteristics and outcomes by reading level.
2. Explore differences in perception of the materials by Intervention or Control group and by reading level.
3. Explore differences in the perception of the tailored and non-tailored material within the Intervention group.

These explorations and analyses will enable us to assess the effectiveness and usefulness of tailoring smoking cession materials to reading level.

## 2 Method

### 2.1 Participants

General Practices in England, Wales, Scotland and Northern Ireland (*n* = 123), selected to be geographically representative of the UK, were recruited from the MRC General Practice Research Framework (MRCGPRF). The study used minimal exclusion criteria. All current cigarette smokers aged 18–65 years were identified from General Practitioner (GP) lists in participating practices. After GP exclusion of patients considered unsuitable for the study (e.g. severe mental impairment, terminally ill), a random sample of 520–550 smokers from each practice (or all smokers in smaller practices) were sent a Smoking Behavior Questionnaire (SBQ) and invitation to participate (*n* = 58,600), Current smokers returning the completed SBQ and signed consent form (*n* = 6911) were enrolled into the study and randomly assigned to the Intervention or Control group. Participants later found to be ineligible (*n* = 214) were excluded leaving 6697 (11.4%) participants at baseline. Recruitment and randomization characteristics are reported in greater detail elsewhere [13].

### 2.2 Baseline measures

The SBQ assessed socio-demographic characteristics, including measures of deprivation, educational level and normal daily reading, intention and motivation to quit smoking, measure of tobacco dependence, and previous quit attempts. Tobacco dependence was calculated based on the Heaviness of Smoking Index [15] and was computed from the number of cigarettes smoked per day and time from waking to first cigarette (score between 0 and 7). An individual deprivation score ranging between 0 and 5 was computed by adding one point for each of the following: renting their home; no car; no educational qualifications; manual occupation; and being unemployed or a full-time student [16].

### 2.3 Interventions

Participants randomized to the Control group received standard, non-tailored information (the NHS ‘Stop Smoking, Start Living’ booklet) [17].

In addition to receiving the standard, non-tailored information, participants randomized to the Intervention group received a computer-tailored advice report. Information obtained from the baseline assessment questionnaire (SBQ) was used to generate the tailored report which also referred to relevant sections in the NHS ‘Stop Smoking, Start Living’ booklet and was accompanied by a very brief letter from the participant's GP endorsing the information in the report.

### 2.4 Reading level (RL)

#### 2.4.1 Reading level of the reports

The advice reports were adapted to two reading levels taking into account design and appearance, readability, layout, font size and color. The Flesch Reading Ease score is a measure of written text comprehension difficulty calculated by combining words per sentence and word length in syllables [18]. It is a popular measure of readability included in Microsoft Office Word, and for ease of assessment, was used to estimate the suitability of the material for the target audience. Scores range from 0 to 100, where higher scores indicate a more easily understood document. In the easy reading version of the report the Flesch score for paragraphs ranged from 77 to 93, averaging 85 which is roughly equivalent to a reading age of 11 years, a sans serif 12-point font (recommended by literacy experts), [19], [20] and used color for emphasis [20].

The standard reading version of the report was written for a general audience, with a Flesch score between 66 and 71, which equates to a reading age of 12–14 years. Thus the standard report was not written beyond the skills of the average reader. Color was also used in the standard report, with a sans serif 10-point font. An example of the easy and standard reading version feedback report is shown in the Supplementary Materials.

#### 2.4.2 Reading level of the booklet

For comparison, the Flesch scores for individual paragraphs in the NHS booklet ‘Stop Smoking, Start Living’ ranged from 55 to 89, on average 71, and the font size ranged from 10 to 13.

#### 2.4.3 Reading level of participants

To assess the reading level of the participant items were selected from a simple screening tool used to identify low literacy levels [21], using the nature of daily reading in terms of newspaper read (i.e. tabloid or broadsheet), and highest qualification. General Certificate of Secondary Education (GCSE) is the first qualification in the British education system, usually completed by students aged 16 at the end of compulsory school education and provides a basic qualification. There is a distinction between UK tabloid and broadsheet newspapers in terms of readability. Mass circulation tabloid papers such as The Sun have higher Flesch scores than broadsheets such as The Times, [22], [23], and preferred daily reading is therefore an indication of the optimum level of reading difficulty. All participants were categorized into the easy reading group (ERG) or standard reading group (SRG). Participants in the Intervention group received the standard reading report unless they fulfilled both criteria of qualifications of GCSE or less and reading a tabloid newspaper. Although participants in the Control group received only standard non-tailored information they were categorized into the ERG or the SRG so that comparisons could be made between reading levels.

### 2.5 Outcome assessment

Follow-up was by postal questionnaire, adapted to a basic reading level to be understood by all participants, sent 6 months after randomization, and by telephone interview for those who failed to return their questionnaire. A full telephone interview was requested if the participant was willing, or, if they were not, two basic outcome measures were asked.

**Fig. 1:**
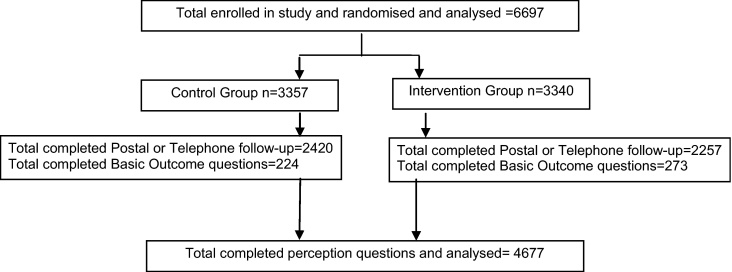
Flow diagram showing participants in the trial and included in the analysis of perception of the materials.

The follow-up response rate based on the initial sample (*n* = 6697) was 77.2% (5174). Follow-up was higher in the SRG than in the ERG (80% vs. 74.9%). The sample analyzed in this paper consists of the 4677 participants (69.8%) who completed either the postal questionnaire or full telephone interview that included the questions on the perception of the materials, excluding 497 participants who gave basic outcome data only (Fig. 1).

### 2.6 Outcome measures

The primary outcome was self-reported prolonged abstinence, defined as no smoking, not even a puff, for at least 3 months at the 6-month follow-up.

The perception outcome measures were similar to ones used in our previous studies [24], [25] and based on Kreuter and colleagues’ [26] recommendations for evaluating tailored health communication programs. They group outcome variables into the following categories: exposure and reading, reaction to appearance, reaction to content, perceived personal relevance, effects on communication with others and perceived usefulness of the information, measured on a five-point Likert-type scale ranging from ‘Not at all’ to ‘Extremely’. Additional outcome measures were added to evaluate the effect of the information on behavior. Examples of the outcome measures used can be seen in Table 1.

### 2.7 Analysis

For categorical variables *χ*^2^ tests were used, and for continuous variables *t*-tests were used to compare baseline characteristics between the two reading levels. To explore differences in the perception of the materials between the Intervention and Control groups and between reading level (RL) *χ*^2^ tests and *t*-tests were used. Logistic regressions with interaction terms for group and RL were used for categorical variables, and for continuous variables General Linear Model was used with interaction terms.

As the Control group received only the NHS ‘Stop Smoking, Start Living’ booklet and the Intervention group received the booklet and the tailored feedback report, group comparisons could only be done for the perceptions of the booklet.

Differences in the perception of the report between RL were analyzed using *χ*^2^ and *t*-tests as before, and to explore the differences in perception of the booklet and the report in the Intervention group only, McNemar and *t*-tests for paired samples were used. This meant that only participants who completed both book and report questions could be included in this analysis, excluding those who did not remember the report and did not answer those questions. To then examine any differences by RL within this group, all cases where, for example, remembering the booklet was not matched by remembering the report, were filtered out for each item, and *χ*^2^ tests carried out for each by RL for the remaining participants i.e. those who remembered only the book or the report. For continuous variables, new variables for the difference in score between perceptions of the booklet and report were calculated and the difference scores compared by RL using *t*-tests.

## 3 Results

### 3.1 Baseline characteristics

Characteristics of participants at baseline by Intervention and Control group are reported elsewhere [13]. Baseline characteristics for the two levels of reading are shown in Table 2. Over half of the sample (53.3%, *n* = 3572) was categorized into the ERG.

The SRG and ERG differed significantly on several baseline characteristics. Participants in the ERG were less likely to be female (53.7% vs. 58.6%, *p* < 0.001), were older (mean age 45.4 vs. 43.7, *p* < 0.001), and had a significantly higher deprivation score (mean 1.97 vs. 0.94, *p* < 0.001). Participants in this group were more nicotine dependent (65.0% vs. 48.3%, *p* < 0.001), and were less likely to have previously quit for 3 months (45.2% vs. 52.9%, *p* < 0.001). Participants in both groups wanted to quit smoking equally (mean 3.3), however ERG participants were less likely to be planning to quit within the next 6 months (37.5% vs. 43.1%, *p* < 0.001) and were more likely to give ‘too difficult’ as the reason for not planning to quit (53.8% vs. 40.8%, *p* < 0.001).

### 3.2 Effectiveness of intervention

The primary outcome, prolonged 3-month abstinence rate, based on intention to treat (i.e. nonresponders at follow-up classed as smokers), was not significantly higher in the Intervention group compared with the Control group (3.2% vs. 2.7% (OR = 1.20, 95% CI [0.94, 1.54], *p* = 0.15)). However, the proportion who reported making a quit attempt in the previous 6 months was significantly higher, (32.3% vs. 29.6% (OR = 1.13, 95% CI [1.01, 1.26], *p* = 0.026)). [13].

Prolonged 3-month abstinence was higher in the SRG than in the ERG overall. However, the relative benefit of the intervention for the primary outcome of prolonged 3-month abstinence was more marked in the ERG. Abstinence rates in the ERG were 2.6% vs. 1.8% (OR = 1.50, 95% CI [0.91, 2.45]) in the Intervention and Control groups respectively, and in the SRG were 4% vs. 3.8% (OR = 1.05, 95% CI [0.78, 1.42]), although the interaction term was not found to be statistically significant (*p* = 0.26).

### 3.3 Perception of booklet by group and by reading level

Significant differences between the Intervention and the Control group were found in their perceptions of the booklet. Participants in the Intervention group were more likely to report reading the booklet (81% vs. 77.9%, *p* < 0.011), keeping the booklet (59.8% vs. 55%, *p* < 0.003), quitting smoking as a result of receiving the booklet (11% vs. 7.9%, *p* < 0.002) and trying to quit smoking as a result of receiving the booklet (37.8% vs. 33.2%, *p* < 0.002). They also found the booklet easier to read (4.27 vs. 4.21, *p* < 0.009), easier to understand (4.30 vs. 4.23, *p* < 0.003), and were more likely to perceive it as written especially for them (2.88 vs. 2.67, *p* < 0.001). Participants in the Intervention group also reported being more confident about quitting (2.53 vs. 2.42, *p* < 0.002) and being more determined to quit (2.59 vs. 2.47, *p* < 0.003) having read the booklet.

The two reading levels differed significantly in their perceptions of the NHS ‘Stop Smoking, Start Living’ booklet. While participants in the SRG were more likely to report the booklet easy to read (4.28 vs. 4.2, *p* < 0.002) and easy to understand (4.31 vs. 4.22, *p* < 0.001), participants in the ERG were more likely to report that the booklet was written especially for them (2.86 vs. 2.68, *p* < 0.001), contained a lot of new information (3.12 vs. 2.64, *p* < 0.001), was interesting (3.54 vs. 3.28, *p* < 0.001) and was useful (3.44 vs. 3.27, *p* < 0.001). They were also, as a result of the booklet, more confident about quitting (2.54 vs. 2.40, *p* < 0.001) and more determined to quit smoking (2.60 vs. 2.46, *p* < 0.001). Perceptions of the booklet by group and by reading level are shown in Table 3.

No interactions were found between RL and Intervention group except for remembering the booklet, where in the SRG more of those in the Intervention group remembered the booklet than in the Control group, this difference was not found in the ERG.

### 3.4 Perceptions of report by reading level (Intervention group only)

Participants in the SRG were more likely to remember receiving the report (71.6% vs. 62.8%, *p* < 0.001) and reading it (85.4% vs. 81%, *p* < 0.02). However, participants in the ERG were in general more positive about the report, and were more likely to consider the advice in the report interesting (3.42 vs. 3.22, *p* < 0.001), useful (3.34 vs. 3.17, *p* < 0.004) and containing new information (3.11 vs. 2.75, *p* < 0.001). Perceptions of the report by reading level are shown in Table 4.

### 3.5 Perception of the booklet vs. report (Intervention group only)

Participants were significantly more likely to report remembering receiving the booklet (94.1% vs. 67%, *p* < 0.001), keeping it (63.8% vs. 58.1%, *p* < 0.001) and discussing it with others (27.7% vs. 25.5%, *p* < 0.01). They were also more likely to try to quit smoking (40.2% vs. 37.1%, *p* < 0.001) as a result of receiving the booklet, in comparison to the report.

While participants reported that the booklet was easier to read (4.30 vs. 4.10, *p* < 0.001), easier to understand (4.33 vs. 4.12, *p* < 0.001), more interesting (3.45 vs. 3.33, *p* < 0.001) and useful (3.43 vs. 3.27, *p* < 0.001), they were significantly more likely to view the report as being written for them (3.16 vs. 2.90, *p* < 0.001), containing more new information (2.94 vs. 2.86, *p* < 0.001), and were more confident (2.66 vs. 2.54, *p* < 0.001) and determined to quit smoking (2.70 vs. 2.60, *p* < 0.001) as a result of reading the report compared to the booklet.

Analysis to examine differences by RL within this group showed that while both reading level groups perceived the report to be more personal than the booklet, the difference was larger for the SRG than for the ERG, although the interaction was not significant at *p* ≤ 0.01 (2.76 vs. 3.10/2.99 vs. 3.23 for the booklet and report respectively) (*p* < 0.028).

## 4 Discussion and conclusion

### 4.1 Discussion

One of the aims of the ESCAPE study was to reach a wide range of smokers of all reading abilities in order to increase cessation in hard to reach groups in low socio-economic and deprived areas. The number of participants recruited with qualifications of GCSE or less demonstrates that the ESCAPE study achieved this objective. Furthermore, comparison of the two reading groups showed that participants in the ERG were living in areas of significantly higher deprivation. The association between RL and deprivation strengthens the evidence for the overlap between social gradient and reading level. This highlights the importance of adapting smoking cessation materials to be appropriate for this hard to reach group of smokers who live in deprived areas and are also likely to have lower literacy levels.

Participants in the ERG and SRG also differed significantly on important baseline variables concerning their dependence and intentions to quit. While their motivation in terms of wanting and being determined to quit was almost identical, participants in the ERG were more dependent and less likely to be planning to quit, more often because it was just too difficult.

The quit rate for the primary outcome of 3 months prolonged abstinence was higher, but not significantly so, in the Intervention group [13]. The quit rate was also higher in the SRG than in the ERG, and while these results reflected the trend for the use of self-help materials to be more successful in higher educated groups [27] the relative benefit of the intervention for the primary outcome was more marked in the ERG. To explore possible reasons for this, perceptions of the booklet and the report by RL were examined to find whether these could have influenced abstinence among participants in the ERG.

Looking at the differences between the two reading levels, on the whole the ERG were more positive in their perceptions of both the booklet and the report. The ERG tended to view the booklet and the report as more interesting and useful, and also perceived them to be more personal than did the SRG. Although the comparison between the SRG and ERG is difficult to interpret because the different versions of the reports were evaluated by groups that differed in reading level and other characteristics, overall these results are encouraging in view of the higher dependence on tobacco among ERG smokers, and suggest that such smokers are receptive to help and encouragement in spite of their apparent reluctance to attempt to quit. Proactively writing to these smokers with individually tailored materials that are attractive and colorful could encourage more individuals to engage in quitting activity. Future tailored reports could perhaps focus on reducing perceptions of difficulty in quitting smoking for this group.

The lack of effect of the intervention in the SRG for prolonged abstinence is perhaps due to the higher effectiveness generally of self-help materials in higher educated smokers [27]. There is also some evidence that questionnaires and assessments alone can prompt self-change by triggering some reflection on behavior [28], and it is possible that this effect was more pronounced in the SRG, where education levels were generally higher.

Comparisons of the booklet and the report in Intervention group participants showed that fewer people remembered receiving the report than the booklet. This is consistent with other studies that found that recipients often neither remember receiving a personal letter nor perceive it as personal [29]. Participants also remembered, kept and discussed the booklet more than the report and found the booklet easier to read, easier to understand, and more interesting and useful than the report. These more positive perceptions of the booklet may be due to the differences in appearance. The booklet was a brochure, similar to other health promotion materials, and produced by the NHS. In comparison the report was written on A4 paper that could be discarded more easily, and participants may have perceived the booklet as more professional and credible. However, participants were more positive about the report in terms of it being more personal. Increased confidence and determination to quit were also reported as a result of reading the report, in comparison to the booklet.

Comparisons between the Control and Intervention group regarding the booklet showed the Intervention group were more positive about the booklet than those in the Control group. They were more likely to read and keep the booklet, to quit or try to quit as a result of the booklet and perceived the booklet as easier to understand and more personally relevant. Intervention participants also reported being more confident and determined to quit as a result of the booklet than Control participants. While the effects are small, these findings suggest the usefulness of personalizing materials simply to bring relevant pages of generic material to the attention of the recipient. Highlighting relevant sections in an accompanying letter appears to make the generic material more memorable and attractive, and while one alone would not be sufficient, both together can work in concert to produce these increases in attention to the communication. In addition participants reported that they felt more confident and more determined to quit smoking as a result of reading the booklet than those in the Control group. This is an important difference, as higher levels of motivation and confidence to quit have been found to be predictive of making a quit attempt [30], and attaching a personal report to a generic booklet could have influenced the increased quit attempt rate in the Intervention group.

A strength of this study is the large sample of smokers, broadly representative of the smoking population across the UK. A sample of this size allows more confidence that the differences found accurately reflect the population it was drawn from. It is important to consider however that the actual differences found are quite small in some cases. Thus, while these differences exist in the population, caution must be applied when considering the practical implications. A further limitation of this study is the use of the proxy measures of educational level and preferred newspaper to assess reading level. Ideally, a longer validated assessment would be used. However the aim of the study was, in line with the principles of computer-tailoring, to deliver a brief tailored letter to a large population of smokers, and this could only be achieved by a postal assessment. The distinction was therefore more pragmatic than theoretical and a practical solution to base the distinction on the smokers’ preferred daily reading, taking in to account their education, was adopted.

While the perception outcome measures were based on recommendations of Kreuter and colleagues [26] for evaluating tailored health communication programs, additional measures assessing perceptions of the credibility and the appearance of the materials in greater depth might increase our understanding of how the materials are perceived. Noar and colleagues [31] suggest that the length of print materials is important as those that are too lengthy may not be read. Views regarding the length, particularly among participants in the ERG, could be useful in the future development of generic and tailored self-help materials.

### 4.2 Conclusion

This brief self-help intervention, designed to reach a wider population of smokers, appeared to have a small but promising effect, which may have been slightly more marked among smokers with a lower level of literacy. On the whole the easier version was well accepted by the group for whom it was intended. Continued work on the adaptation of materials to reading level is warranted. However, further research is needed in the form of a mismatched trial where participants categorized as both easy and standard reading are randomized to receive either an easy or a standard reading report, to assess whether smoking cessation advice matched to reading level is more effective than non-matched.

### 4.3 Practice implications

The social gradient and overlap between those who have low reading ability and fewer qualifications and smoking, and the significant differences by reading level in dependence and intentions to quit, emphasize the need to adapt smoking cessation materials to address the needs of smokers who live in deprived areas and who are also likely to be of a lower level of literacy. While layout and appearance of material is important, the perception of the information as personal and relevant to the needs of the recipient is also critical in bringing written materials to the attention of the reader.

## Funding

This study was funded by Cancer Research UK (Grant Number C16265/A6907).

## Conflict of interest

None declared.

## Figures and Tables

**Table 1:** Perception outcome measures and scales used.

Category	Measure	Scale
Exposure and reading	• ‘Do you remember receiving this booklet’ • ‘Have you read all of the booklet’ • ‘Have you kept the booklet’	Yes/No
Reaction to appearance	• ‘I liked the appearance of the booklet’	5-point Likert scale: Not at all – Extremely
Reaction to content	• ‘The booklet was easy to read’ • ‘The booklet was easy to understand’ • ‘The advice in the booklet was interesting’ • ‘The booklet contained a lot of new information’ • ‘I liked the tone of the report’^a^	5-point Likert scale: Not at all – Extremely
Perceived personal relevance	• ‘The booklet was written especially for me’	5-point Likert scale: Not at all – Extremely
Effects on communication with others	• ‘Have you discussed the booklet with others’	Yes/No
Perceived usefulness of the information	• ‘The advice in the booklet was useful’ • ‘As a result of the booklet I am more confident about quitting’ • ‘As a result of the booklet I am more determined to quit’	5-point Likert scale: Not at all – Extremely
Effects on behavior	• ‘As a result of receiving the booklet: Have you quit smoking? Have you tried to quit smoking? Do you intend to quit smoking?’ • ‘Have you completed the lists on the sheet at the end of the report?’^a^	Yes/No

a All perception outcome measures were asked in relation to both the booklet and the report, with the exception of two additional outcome measures for the report: ‘I liked the tone of the report’ and ‘Have you completed the lists on the sheet at the end of the report’.

**Table 2:** Characteristics of participants at baseline by reading level.

	Reading level				Total (*n* = 6697)		*p*
	ERG 53.3%/(*n* = 3572)		SRG 46.7%/(*n* = 3125)				
% Intervention group	1804	(50.5)	1536	(49.2)	3340	(49.9)	0.27
% Female	1918	(53.7)	1830	(58.6)	3748	(56.0)	<0.001
Mean age (SD)	45.4	(12.1)	43.7	(12.3)	44.6	(12.2)	<0.001

Qualifications							
% None	1729	(48.4)	96	(3.1)	1825	(27.5)	
% GCSE	1843	(51.6)	543	(17.7)	2386	(36.0)	
% A Level	0	(0)	1188	(38.8)	1188	(17.9)	
% Degree	0	(0)	985	(32.2)	985	(14.9)	
% Postgraduate	0	(0)	248	(8.1)	248	(3.7)	<0.001

Mean cigarettes per day (SD)	19.3	9.7	16.0	8.8	17.7	9.4	<0.001
% Smoked within 30 minutes of waking	2476	(69.6)	1674	(53.8)	4150	(62.2)	<0.001
% Previously quit for more than 3 months	1612	(45.2)	1651	(52.9)	3263	(48.8)	<0.001
% Non-daily smokers	112	(3.2)	232	(7.5)	344	(5.2)	<0.001

Dependence category^a^							
%Low	378	(10.7)	656	(21.2)	1034	(15.6)	
%Medium	862	(24.3)	945	(30.5)	1807	(27.2)	
%High	2303	(65.0)	1497	(48.3)	3800	(57.2)	<0.001

Intentions to quit							
%Within next 2 weeks	170	(4.8)	158	(5.1)	328	(4.9)	
%Within next 30 days	284	(8.0)	241	(7.7)	525	(7.8)	
%Within next 6 months	1340	(37.5)	1348	(43.1)	2688	(40.1)	
%Not within next 6 months	1778	(49.8)	1378	(44.1)	3156	(47.1)	<0.001

If not planning to quit, why not?							
%Too difficult	902	(53.8)	532	(40.8)	1434	(48.1)	
%Want to smoke	699	(41.7)	711	(54.4)	1410	(47.3)	
%Both	75	(4.5)	61	(4.7)	136	(4.6)	<0.001

Mean Score ‘How much do you want to quit for good?’ (scale 1–5) (SD)	3.3	(1.2)	3.3	(1.1)	3.3	(1.2)	0.72
Mean score ‘How determined are you to quit for good? (scale 1–5) (SD)	3.2	(1.2)	3.2	(1.2)	3.2	(1.2)	0.54
% Living with an adult smoker	1422	(39.9)	1188	(38.1)	2610	(39)	0.14
Mean deprivation score (0–5) (SD)^b^	1.97	(1.35)	0.94	(1.06)	1.48	(1.32)	<0.001

a Dependence score was computed from the number of cigarettes per day and time from waking to first cigarette.

b Deprivation score was computed by scoring one point for each of the following: renting their home, no car, no qualifications, manual occupation and being unemployed or student.

**Table 3:** Perception of the booklet by Intervention and Control Group and by Reading Level (ERG and SRG).

Group	Control group		Intervention group		All	Comparison between Control and Intervention group	Comparison between ER and SR levels	Interaction between intervention and reading level
Reading level	ERG	SRG	ERG	SRG	*n*/mean	*p*	*p*	*p*
	*n*/mean	*n*/mean	*n*/mean	*n*/mean				
*n*(%) Remember receiving	1137 (93.8)	1110 (92.5)	1093 (93.3)	1024 (95)	4364 (93.6)	0.18	0.89	0.04
*n*(%) % Read all	907 (77.2)	925 (78.7)	903 (80.2)	864 (81.8)	3599 (79.4)	0.011	0.22	0.89
*n*(%) Kept	667 (57.3)	625 (53.7)	664 (59.7)	628 (60)	2584 (57.6)	0.003	0.23	0.21
*n*(%) Discussed with others	268 (23.1)	276 (23.7)	277 (24.8)	260 (24.9)	1081 (24.1)	0.25	0.80	0.83
*n*(%) Quit as a result of	69 (7.6)	74 (8.1)	86 (10.4)	91 (11.6)	320 (9.3)	0.002	0.42	0.85
*n*(%) Tried to quit as a result of	347 (32.8)	338 (33.6)	388 (39.5)	320 (36)	1393 (35.4)	0.002	0.39	0.16
*n*(%) Intend to quit as a result of	677 (64.2)	676 (66)	622 (63.1)	586 (65)	2561 (64.6)	0.47	0.23	1.0
Mean (SD)score ‘Easy to read’ (scale 1–5)	4.16 (0.82)	4.25 (0.76)	4.24 (0.82)	4.30 (0.73)	4.24 (0.79)	0.009	0.002	0.6
Mean score ‘Easy to understand’ (scale 1–5) (SD)	4.18 (0.78)	4.28 (0.72)	4.27 (0.78)	4.34 (0.70)	4.27 (0.75)	0.003	<0.001	0.56
Mean score ‘Written especially for me’ (score 1–5) (SD)	2.74 (1.22)	2.61 (1.17)	2.99 (1.24)	2.76 (1.19)	2.77 (1.21)	<0.001	<0.001	0.16
Mean score ‘Contained a lot of new information’ (scale 1–5) (SD)	3.13 (1.10)	2.62 (1.11)	3.11 (1.18)	2.66 (1.12)	2.89 (1.15)	0.64	<0.001	0.40
Mean score ‘Advice was interesting’ (scale 1–5) (SD)	3.52 (1.0)	3.26 (1.02)	3.57 (1.03)	3.31 (1.03)	3.41 (1.03)	0.09	<0.001	0.97
Mean score ‘Advice was useful’ (score 1–5) (SD)	3.40 (1.11)	3.23 (1.09)	3.48 (1.09)	3.30 (1.09)	3.36 (1.10)	0.02	<0.001	0.85
Mean score ‘Like the appearance’ (score 1–5) (SD)	3.33 (0.98)	3.30 (0.92)	3.36 (1.02)	3.30 (0.99)	3.32 (0.98)	0.57	0.16	0.57
Mean score ‘As a result I feel more confident about quitting’ (scale 1–5) (SD)	2.49 (1.20)	2.34 (1.14)	2.59 (1.23)	2.46 (1.2)	2.47 (1.2)	0.002	<0.001	0.95
Mean score ‘As a result I feel more determined to quit’ (scale 1–5) (SD)	2.54 (1.27)	2.40 (1.21)	2.66 (1.33)	2.51 (1.22)	2.53 (1.26)	0.003	<0.001	0.79

**Table 4:** Perception of the report by reading level (Intervention group only).

	Reading level		All	*p*
	ERG	SRG	*n* = 2257	
	*n* = 1175	*n* = 1082		
*n*(%) Remember receiving	727 (62.8)	768 (71.6)	1495 (67.0)	<0.001
*n*(%) Read all	608 (81.0)	675 (85.4)	1283 (83.3)	0.02
*n*(%) Kept	428 (58.0)	455 (58.2)	883 (58.1)	0.96
*n*(%) Discussed with others	178 (24.0)	207 (26.4)	385 (25.2)	0.32
*n*(%) Quit as a result of	58 (10.0)	79 (13.3)	137 (11.7)	0.08
*n*(%) Tried to quit as a result of	253 (38.4)	229 (35.8)	482 (37.1)	0.33
*n*(%) Intend to quit as a result of	418 (63.8)	429 (65.8)	847 (64.8)	0.49
Mean score ‘Easy to read’ (scale 1–5) (SD)	4.07 (0.85)	4.13 (0.79)	4.10 (0.82)	0.15
Mean score ‘Easy to understand’ (score 1–5) (SD)	4.09 (0.84)	4.16 (0.76)	4.13 (0.80)	0.08
Mean score ‘Written especially for me’ (scale 1–5) (SD)	3.23 (1.23)	3.10 (1.22)	3.16 (1.23)	0.051
Mean score ‘Contained a lot of new information’ (scale 1–5) (SD)	3.11 (1.09)	2.75 (1.06)	2.92 (1.09)	<0.001
Mean Score ‘Advice was interesting’ (scale 1–5) (SD)	3.42 (1.04)	3.22 (1.04)	3.32 (1.04)	<0.001
Mean score ‘Advice was useful’ (scale 1–5)	3.34 (1.11)	3.17 (1.10)	3.26 (1.11)	0.004
Mean score ‘I liked the tone of the report’ (scale 1–5) (SD)	3.40 (1.00)	3.36 (1.00)	3.38 (1.00)	0.43
Mean scale ‘Like the appearance’ (scale 1–5) (SD)	3.21 (0.95)	3.15 (0.96)	3.18 (0.96)	0.31
Mean score ‘As a result I feel more confident about quitting’ (scale 1–5) (SD)	2.68 (1.25)	2.61 (1.22)	2.64 (1.24)	0.26
Mean score ‘As a result I feel more determined to quit’ (scale 1–5) (SD)	2.72 (1.29)	2.63 (1.27)	2.67 (1.28)	0.17
*n*(%) Completed lists	172 (24.4)	185 (24.2)	357 (24.3)	1.00
