# Supplementary material for: Computer-tailored smoking cessation advice matched to reading ability: Perceptions of participants from the ESCAPE trial
Source: Patient Educ Couns. 2015 Dec;98(12):1577–84. doi: 10.1016/j.pec.2015.06.013 (PMC4655864; doi:10.1016/j.pec.2015.06.013)
Supplement: Supplementary file 1 [file mmc1.docx]

**SUPPLEMENTARY MATERIAL
EXAMPLE OF A EASY READING GROUP ADVICE REPORT**

PQA

**Personal Quit Advice for Jane Smith**

**You have decided to quit.** Congratulations! You should be proud of yourself. This report is written for you personally to give you extra help. It is based on your answers in the questionnaire you filled in recently. The logo
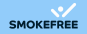
 in this report refers to the ‘STOP SMOKING START LIVING’ booklet enclosed. We hope it will help you to think about your smoking, and boost your confidence by giving you things that you can do when you are tempted to smoke.

You told us you were planning to quit on 01 January 2005, so you may not have smoked since then. If so, well done. You want to quit and are determined. These things are important and show that you are prepared to put effort into it. If you haven’t stopped yet, or if you stopped and then had another cigarette, don’t feel bad. Setting a quit date is a good thing, and gives you something to work towards. So:

- If you have quit and not smoked since, that’s brilliant. Restate your decision and keep it up.
- If you are still smoking, remember it’s never too late. Set another date, make sure you are ready and make your mind up to go through with it.

**Your reasons for quitting**

Your main reason to quit is for your health, and smoking is already damaging your health. You may know the major health risks of smoking. But don’t let your health problems worry you more. Just **remind yourself that quitting is the single most important change you can make.** When you stop smoking, your health will begin to improve at once. The sooner you stop the quicker it will improve.


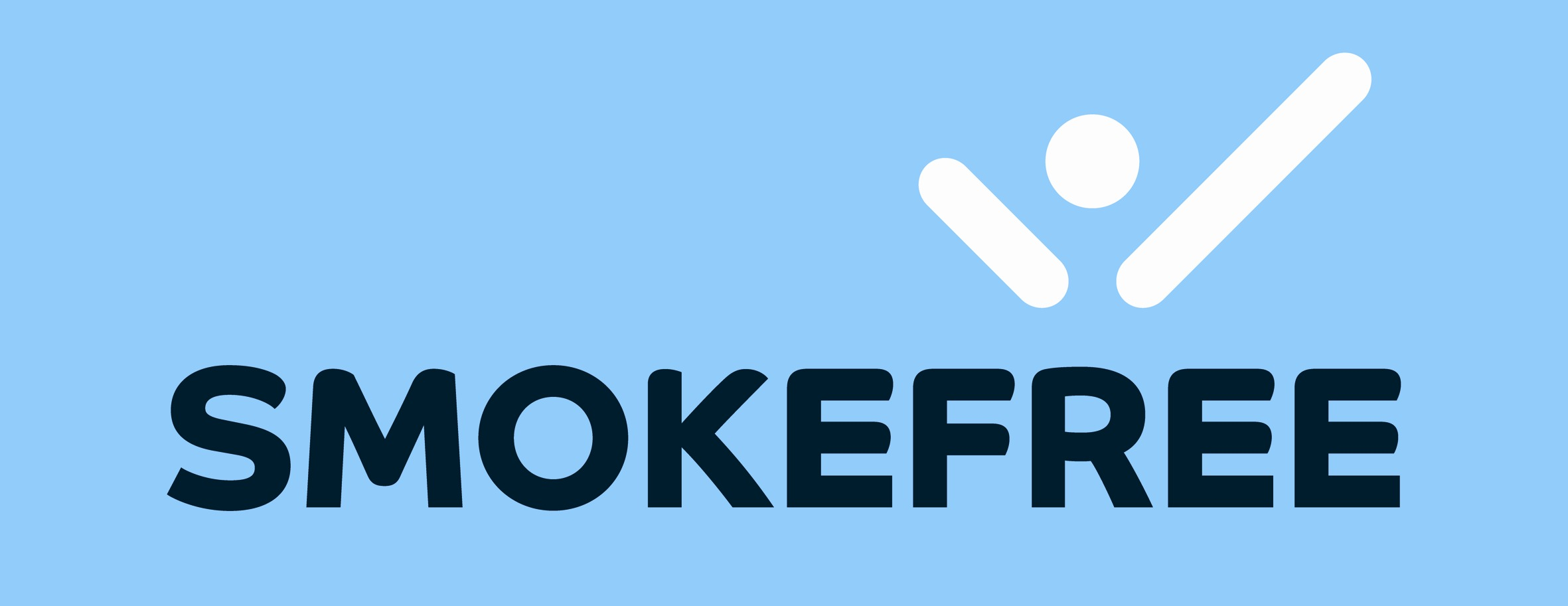
 *Read more about the benefits of quitting on page 9*

*or go to* [*www.gosmokefree.co.uk*](http://www.gosmokefree.co.uk) *to find out more about the risks of smoking*


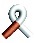
When you quit it will help your children too. Passive smoking can cause childhood asthma, bronchitis and cot death. Children copy their parents; so older children will be less likely to smoke if you quit.

*Learn more about how you can help your children in the booklet ‘P is for protecting babies and children from secondhand smoke’.*

*Write down on the attached sheet why you want to quit, and keep reminding yourself of it.*


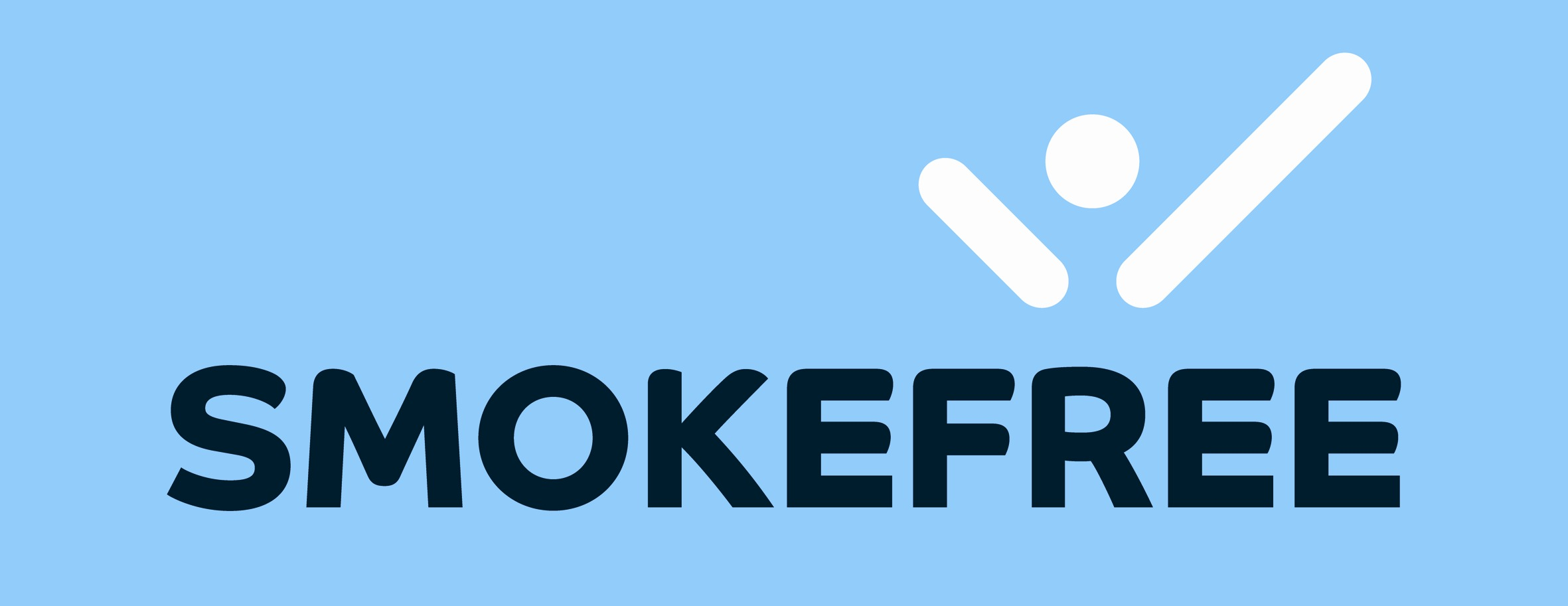
 *Use the list on pages 27 and 29*

*
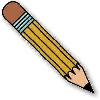
*


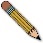
Don’t forget as well how much money you can save by quitting. You said that you smoke 30 cigarettes a day. By quitting, you will save about

*Write down 2 things you would like that you could buy with this money*


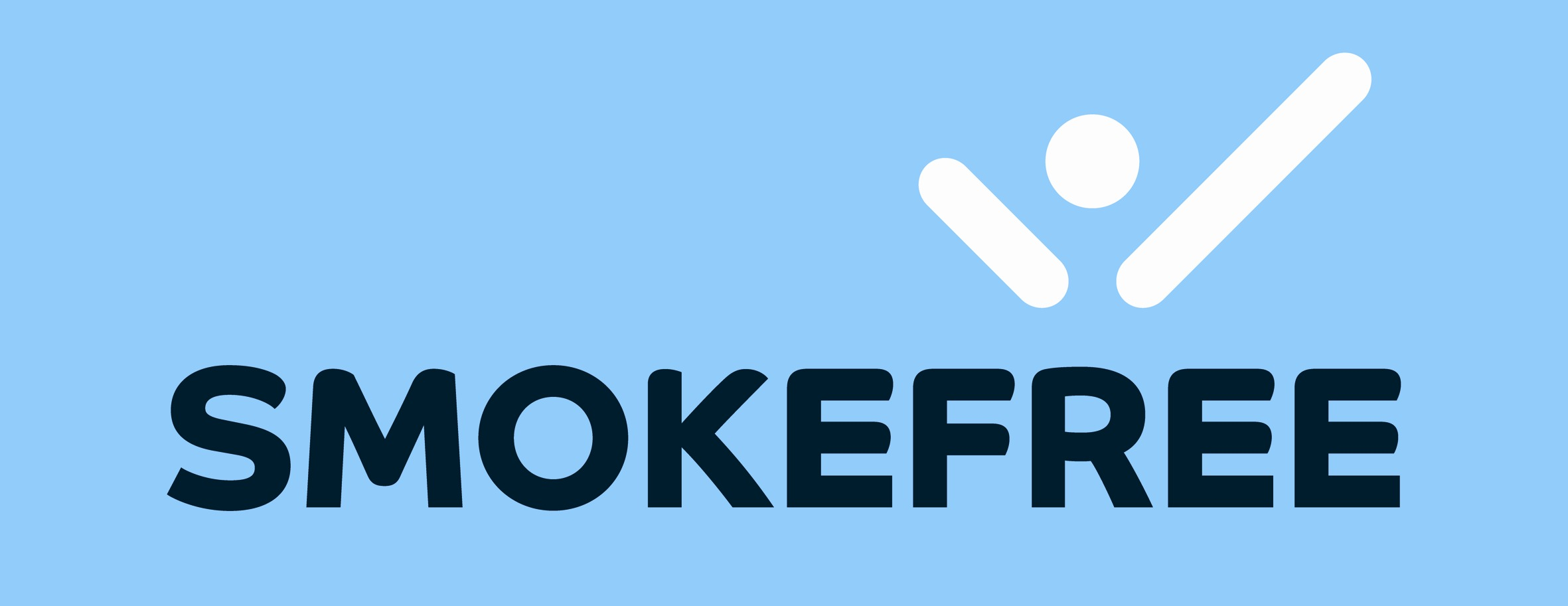
 *Work it out for yourself page 27*

- £57 per week
- £2964 in a year.

Think what you can do with this money, and you’ll enjoy these treats more with better health.

**Changing your self-image**

You think you are addicted to smoking. But you don’t see the habit as part of your personality, and that’s good. It is the nicotine in your brain telling you that you need it. Your task is to beat these thoughts.

- tell yourself you can beat them
- tell yourself that you don’t need the nicotine.

You can already see yourself as a non-smoker, that’s good too.

- keep calling yourself a non-smoker and increase this belief.

If you’ve tried to quit before, don’t be put off. Learn from the past and plan different things to do this time.

At your level of smoking, we suggest that you use one of the products that will help you through the early days while you break the habit. They come in different forms. Talk to your GP or practice nurse about it, and find the one that suits you best. Be determined and stick with it, and the craving will get less over time.

**Quitting is a positive step**

You are worried about feeling dull and bored if you stop smoking. We can understand this. But think about all the smokers who have given up and still enjoy life. Remember that it is just a habit. You can break it if you find other, healthier things to do. Take up a hobby, something that will occupy your hands. You will improve your health and add some other interest to your life.

**For when you may be tempted**

The hardest time not to smoke is different for everyone. For some people it is with other smokers. For others, when they get an urge or craving to smoke, or when they are feeling angry or stressed. For others when they are relaxing, alone or with friends. Think about the time that is most difficult for you, and plan things to do. Talk to ex-smokers or non-smokers and find out how they feel, and how they manage not to smoke. Think carefully about the times you get angry and stressed. And whether smoking will make the problem go away. There are other ways of coping. Try to break the link in your mind between places and smoking.

There is not just one way to deal with the temptation to smoke. Try different things to see what works for you. Work out ahead of time things you will do and practise doing them.

Here are some tips for you to try:

- Picture yourself having fun without smoking when you are with friends.
- Practise telling people that you don’t smoke.
- Practise saying no to a cigarette.
- Change your routine, to break link with smoking.
- If you feel tempted, get out of the room for a while.
- Put some space between yourself and the cause of the stress.
- Go to non-smoking areas in public places.
- Have something in your hands to fiddle with.
- Think about the effect on your resolve to quit of having a cigarette.
- Practise deep breathing to help you relax.
- Take a walk, do some exercises.
- Remind yourself that smoking doesn’t really help solve your problems.
- Put the money you save in a box or jar and buy yourself a treat.

Think about times when you are confident and you can resist smoking. Use this confidence at times when you are tempted to smoke.

##### Write down 2 things that you are going to do when you are tempted

*
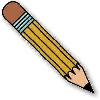
*

**Don’t go it alone**

You live with other people who plan to quit too. That’s great! Make the most of it. Quitting with someone else can make it easier. Use the support from your family and friends too. Ask them to encourage you and thank them for helping. But don’t just rely on friends. Quitting is up to you, and it will be your success.

If you want to talk to someone, ring

the Quitline on 0800 002200

or your surgery

or your local stop smoking service, Cignificant on 0800 0854 113

Congratulations on your decision to quit. It is one of the best decisions of your life. Plan ahead and use the skills you’ve learnt. Stay on track and keep a positive outlook. Remember quitting smoking is not a loss, but a gain.

**Good Luck! and Remember**

**You Can Do It**

**______________________________________________________________**

The **Personal Quit Advice** program was developed by Health Psychologists and smoking cessation experts at University College London and the University of Cambridge in collaboration with QUIT.

**Use this sheet to make your lists. Then pin it up somewhere to remind you.**

I want to quit because:

_______________________________________________________

_______________________________________________________

_______________________________________________________

Two things that I want to buy with the money I save are:

1)________________________________________

2)________________________________________

Next time I am tempted to smoke I will:

1)________________________________________

2)________________________________________
