# Supplementary material for: Computer-tailored smoking cessation advice matched to reading ability: Perceptions of participants from the ESCAPE trial
Source: Patient Educ Couns. 2015 Dec;98(12):1577–84. doi: 10.1016/j.pec.2015.06.013 (PMC4655864; doi:10.1016/j.pec.2015.06.013)
Supplement: Supplementary file 2 [file mmc2.docx]

**SUPPLEMENTARY MATERIAL**

**EXAMPLE OF A STANDARD READING GROUP ADVICE REPORT


Personal Quit Advice for Jane Smith**

PQA

**You have made the decision to quit in the next 6 months.** Congratulations! This is a positive step and you deserve to feel proud of yourself. This report is intended for you personally, based on your answers in the questionnaire that you completed recently. The logo
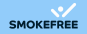
 in the report refers to the ‘STOP SMOKING START LIVING’ booklet that is also enclosed. We hope you find these useful, and that they will help you to become aware of your thoughts and attitudes towards your smoking. We also hope that it will increase your confidence in your ability to quit successfully.

You may not be ready to quit immediately, but that’s okay. Quitting smoking involves changes in thinking before you are ready to take action, and you need time to plan ahead and be prepared. We suggest that you think carefully about your reasons for smoking, and about when and where you smoke. Then develop a plan for this positive change that fits in with your lifestyle and routines. When you are fully prepared to carry out your plan, set a quit date, it will help you to make a commitment. Your desire to stop and determination are high. That is excellent, these are important to success, you are well on your way to becoming smoke free.

**Your reasons for quitting**

Your main reason to quit would be because you are concerned about health, and you are right to be concerned. You may be aware of the major health risks of smoking. But did you know of the many other diseases caused by smoking?


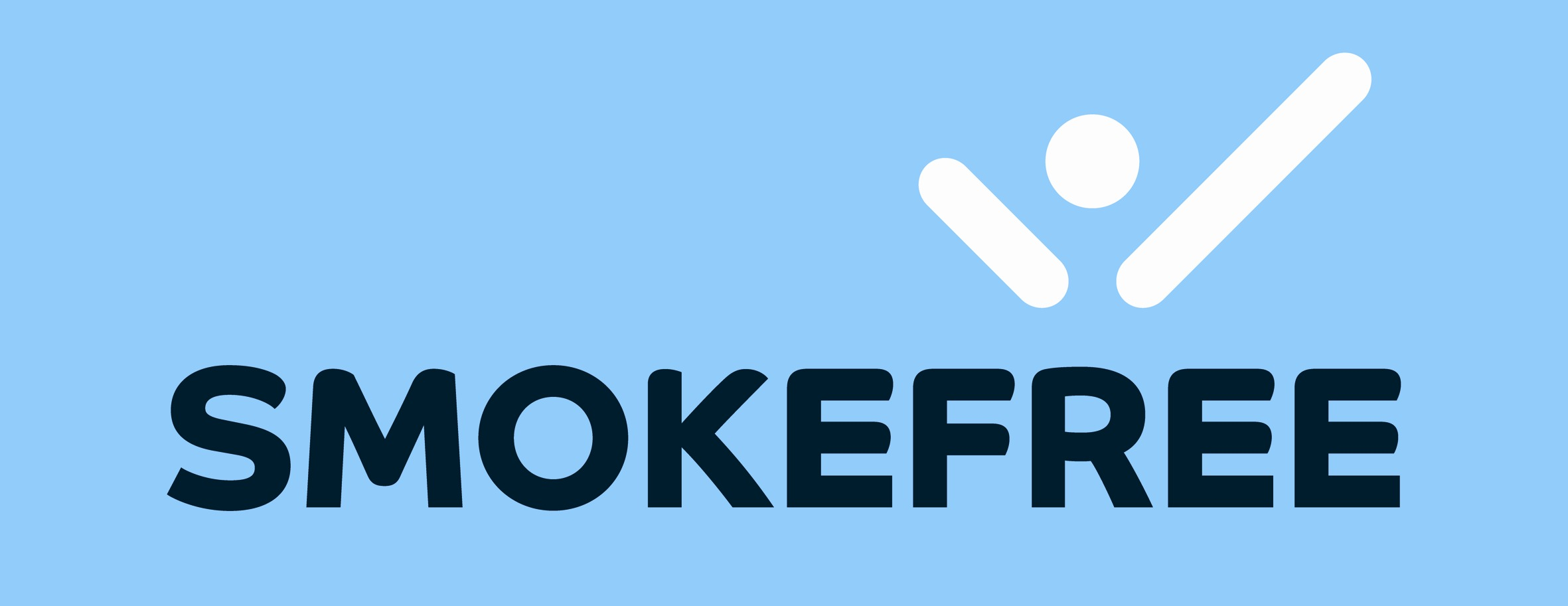
 *Read more about the benefits of quitting on page 9*

*or go to* [*www.gosmokfree.co.uk*](http://www.gosmokfree.co.uk) *to find out more about the risks of smoking*

**Risks of smoking:**

- heart disease and stroke
- cancer of the lungs, mouth, throat, stomach, liver, bladder, bowel, uterus, ovary, cervix, breast
- chronic lung disease (emphysema and bronchitis)
- ulcers
- tooth loss and gum disease
- loss of sight
- fertility problems
- early menopause
- wrinkles and clogged pores
- yellow teeth
- death in middle age

These risks are 10 times greater if you are taking the pill


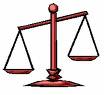


**Benefits of quitting:**

- improved circulation
- immediate lower risk of fatal blood clots
- risk of heart disease halved in one year
- reduced risk of developing cancer
- oxygen levels rise and
  - feel more alert
  - increased energy level
  - better able to walk and exercise
- tar and debris is cleared from lungs
- coughing and wheezing is reduced
- breathing is improved
- fewer chest infections
- quicker healing of wounds
- sharper sense of taste and smell
- fresher smelling clothes
- skin is able to breathe and looks better
- longer life and better overall quality of life

The link between smoking and these illnesses is not so well known, so you may underestimate the overall harmful effect of smoking. Try to learn more about the risks of smoking and how dangerous it is to you personally. Think carefully about whether you value smoking more than your health. Although you may not have a health problem yet, you can’t afford to be complacent, because you may already have unseen damage. Heart disease, cancer and emphysema may be developing for years before you become aware of it. Remember that quitting smoking is the single most important change you can make, and it is never too early to act to start reversing any damage and reduce your risk of severe health problems.

You might also consider how your smoking affects other important people in your life, especially your children. Passive smoking can cause smoking related illnesses such as stroke, as well as childhood complaints such as asthma, and is directly related to cot deaths. Children breathe faster than adults and are particularly at risk from the harmful products in the air. Older children are more likely to smoke if they see parents smoking, so by stopping you would be sending a positive message to your children.

*The booklet ‘P is for protecting babies and children from secondhand smoke’ will give you more information to help you to do the best for your children.*

*Write down on the attached sheet some reasons why you think you might quit.*


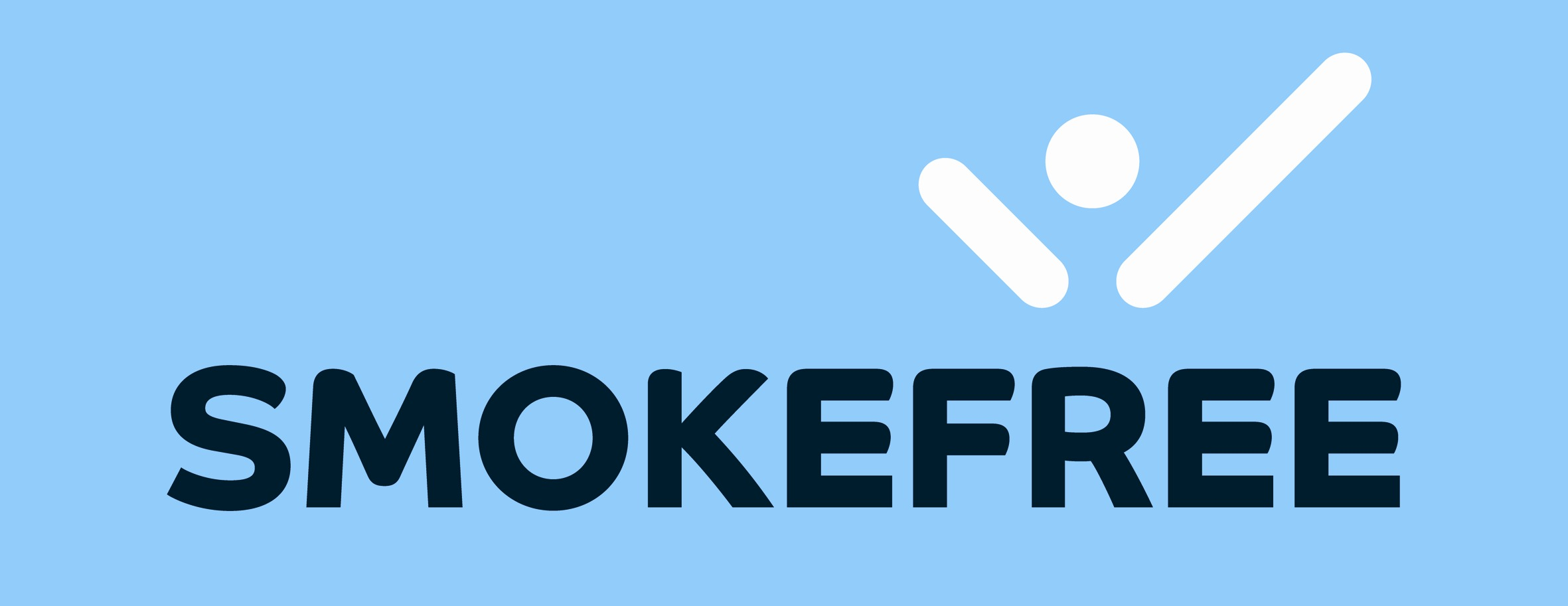
 *Use the lists on pages 27 and 29 to help you decide*

*
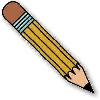
*


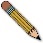
Don’t forget also the amount of money you can save by quitting. You said that you smoke 25 cigarettes a day, which must cost you about £48 per week. By quitting, in one year you would save £2496, in five years £12480. Think what you could spend this money on if you didn’t smoke.

*Write down 2 things you would like that you could buy with this money*


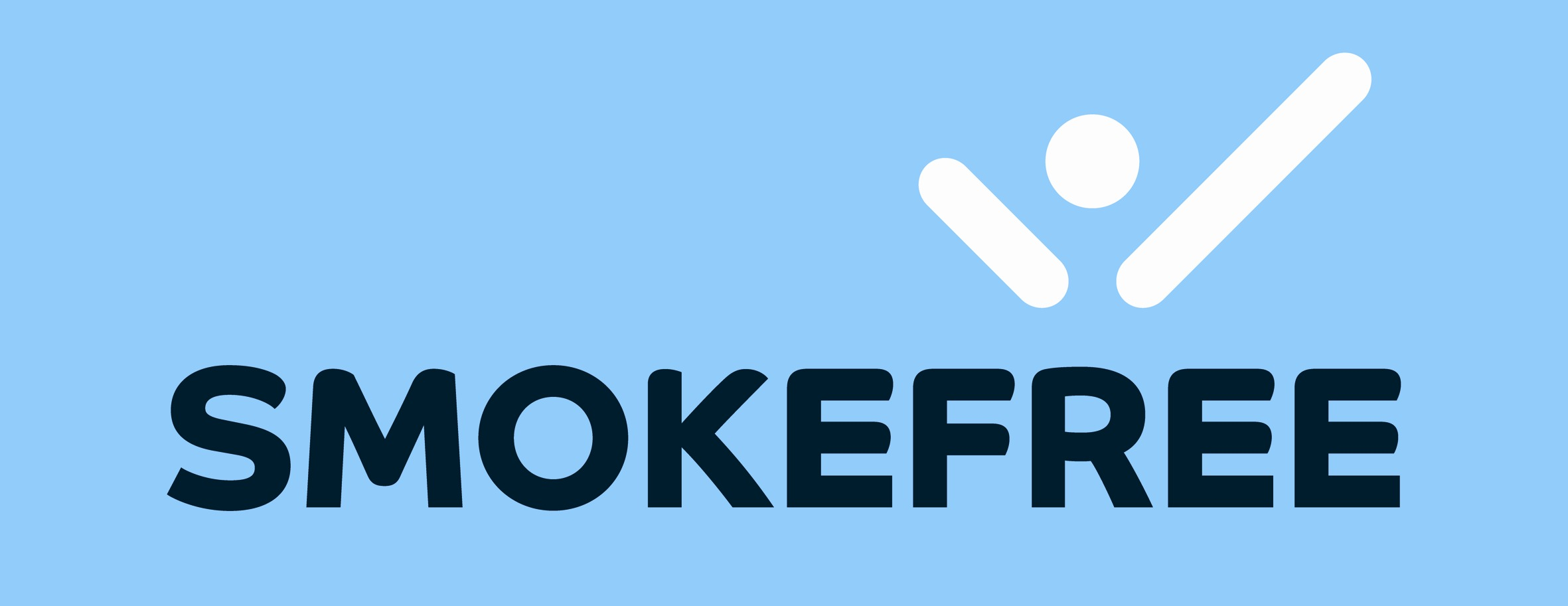
 *Work it out for yourself page 27*

**Changing your self-image**

You see yourself as addicted to smoking, but you don’t see smoking as part of your personality. This shows that you already think of yourself separately from the habit. Your challenge is to overcome the messages from your brain telling you that you need nicotine. Confront these thoughts and tell yourself that you can overcome them and you don’t need the nicotine. You can see yourself as a non-smoker, so keep calling yourself a non-smoker, practise saying it and promote this belief. If you have previously tried to quit, don’t be discouraged. Learn from your past experiences and plan new ways of coping next time.

At your current level of smoking there are products that you can use which will increase your chances of success, and we recommend that you use one of these. The products come in many different forms, talk to your GP about it and find the product that suits your lifestyle. It will help you through the early days while you are breaking free of the habit. Be determined and stick with it, and the craving will diminish over time.

**Quitting is a positive step**

You are worried about feeling tense and irritable when you stop smoking. This is understandable, but any tension and frustration is just the result of you not doing something that you have previously enjoyed. People can feel bad for lots of reasons, and it is possible that your bad mood is not related to smoking. Cigarettes may appear to make life more manageable but are really a false way of coping. They build up the stress so that you think you need to smoke to relieve it, in reality quitting will lead to you feeling less stressed and irritated, improving both your mental and physical health.

Think carefully about the advantages and disadvantages of quitting. If you approach quitting as a positive move, and believe that the benefits of quitting are more important than the hardships, you will feel good about not smoking and about yourself.

##### Make a list of the advantages and disadvantages of quitting

*
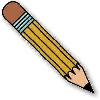
*

**Increase your confidence**

Most people have a particular place or time when they find it more difficult not to smoke. Smoking is a learned response to certain situations or activities and moods. Your mind associates negative emotions, feeling angry or stressed, with smoking. But smoking is just a distraction, there are other distractions and ways of coping.

You can prepare for quitting by trying different ways to practise breaking the association, and dealing with negative feelings without smoking. Relax, do some deep breathing, focus on another task or leave the situation for a few minutes to distance yourself from the source of the stress, take a walk, do some exercises, or do anything to take your mind off it. Ask yourself whether smoking will make the problem go away.

*Take some small steps to increase your confidence and to see what works for you. What will you do today?*

**Don’t go it alone**

You may not be sure of support from your family and friends, but don’t let it put you off. Use it as extra motivation, show them that you can do it without their help, and maybe your success will encourage them to quit too. When you are out, avoid smokers who might demotivate you. But don’t attempt to do it all by yourself; try to find a person that you can trust to act as a support and tell them that you appreciate them listening and helping at a difficult time.

Don’t forget that if you need someone to talk to, you can ring the Quitline on 0800 002200. You can also get help from local health professionals at your surgery, or by ringing All Wales Stop Smoking Service on 0800 085 2219.

Finally, your decision to quit is one of the best decisions of your life. Plan ahead and prepare for when you quit. Keep this letter, return to it and read it later when you are ready. Remember smoking does not solve your problems, and quitting is not a sacrifice, but a gain. There is nothing to be lost from quitting smoking, but everything to gain.

**Good Luck!**

**Remember You Can Do It**

**______________________________________________________________**

The **Personal Quit Advice** program was developed by Health Psychologists and smoking cessation experts at University College London and the University of Cambridge in collaboration with QUIT.

**Use this sheet to make your lists. Then pin it up somewhere to remind you.**

My reason to quit is:

_______________________________________________________

_______________________________________________________

_______________________________________________________

Two things that I could buy with the money I save are:

1)________________________________________

2)________________________________________

Advantages of quitting Disadvantages of quitting

________________________ ____________________

________________________ ____________________

________________________ ____________________

________________________ ____________________

________________________ ____________________

________________________ ____________________

________________________ ____________________

*Which list is longer?*

Today I will:

_______________________________________________________

_______________________________________________________
